# Trust, but Verify—Post-Hoc Analysis of Industrial Machine Learning via Interpretability Metric Embedding and Surrogate Mapping

**DOI:** 10.3390/s26103232

**Published:** 2026-05-20

**Authors:** Simon Mählkvist, Pontus Netzell, Thomas Helander, Konstantinos Kyprianidis

**Affiliations:** 1 Kanthal AB, SE-734 27 Hallstahammar, Sweden; 2 Future Energy Center, Mälardalen University, SE-721 23 Västerås, Sweden

**Keywords:** post-hoc analysis, explainable AI, UMAP, interpretability metrics, industrial machine learning, decision landscape

## Abstract

In industrial machine learning, predictive performance alone is insufficient to ensure reliable deployment, as model behaviour may vary across different regions of the input space under limited data and evolving process conditions. This work investigates whether such variation can be systematically analysed through post-hoc methods. A model-agnostic framework is proposed in which interpretability metrics, including residuals and feature attributions, are embedded into a low-dimensional space and approximated using a continuous surrogate model. This representation enables the analysis of model behaviour as a structured landscape, rather than as isolated pointwise explanations. The approach is applied to ceramic heating element production, where two distinct regimes are identified. One corresponds to a stable region with consistent and accurate predictions, while the other reflects a transitional regime associated with increased ambiguity and sensitivity to feature interactions. These regimes are shown to align with known process conditions and temporal variation. The results demonstrate that model behaviour can be organised into coherent regions that are not observable through aggregate performance metrics alone. This provides a structured basis for post-hoc analysis, supporting targeted interpretation and further investigation of model reliability in industrial settings.

## 1. Introduction

Transparency in how machine learning models behave and produce predictions has been recognised as a central concern within industrial contexts [1]. An understanding of when and why a model may fail is often considered equally important as achieving high predictive accuracy, particularly within safety-critical or economically sensitive manufacturing environments [2]. Despite these requirements, modern machine learning models are frequently treated as black-box systems, limiting their ability to provide explanations aligned with underlying system behaviour [3]. Consequently, there exists a pressing need for analytical methodologies capable of exposing model blind spots, quantifying uncertainty, and providing explanations that are both trustworthy and actionable. Such methods ultimately foster trust among domain experts by increasing the transparency of model reasoning, thereby supporting decision-making under uncertain conditions [4].

In practice, the deployment of machine learning models in industrial settings is often constrained by limited data availability, pronounced class imbalance, and insufficient coverage of rare but critical cases. These conditions frequently result in incomplete model representations and reduced reliability when applied in operational environments [5]. Moreover, industrial processes are characterised by concept drift, wherein the statistical relationships between inputs and outputs evolve over time. This phenomenon can significantly degrade model performance unless explicitly monitored and mitigated [6]. Complementary work by [7] provides a formal characterisation and taxonomy of drift types, underscoring the importance of robust diagnostics in non-stationary industrial settings. As such, conventional data-driven modelling approaches, which typically depend on large, balanced, and diverse datasets, are often ill-suited for these evolving scenarios. Consequently, the post-hoc analysis of trained models must contend with limited sample availability, shifting data distributions, and the risks of overfitting or overgeneralisation, necessitating robust and adaptive interpretive methods.

Recent work has combined Digital Twin models with transfer learning to enable data-driven fault prediction under limited data conditions [8]. However, such approaches primarily emphasise predictive accuracy rather than structured analysis of model behaviour.

Cost-sensitive learning has previously been adopted to manage the trade-off between predictive performance and operational risk, offering a pragmatic route to risk-aware decision support in batch processing environments [9]. Nevertheless, such scalar thresholds provide only a coarse understanding of model reliability. To address these limitations, recent approaches have explored post-hoc, model-agnostic frameworks that integrate interpretability metrics with low-dimensional embeddings [10]. These frameworks are intended to uncover localised process structures and support explainable risk transparency.

Ribeiro et al. [11] introduced LIME, a model-agnostic technique designed to approximate local decision boundaries. This approach enables individual predictions to be interpreted without requiring access to the model’s internal structure. Building on this, Lundberg and Lee [12] proposed Shapley Additive exPlanations (SHAP), a unified framework based on cooperative game theory, which provides consistent, additive feature attributions across different model classes. Barredo Arrieta et al. [10] provided a comprehensive review of explainable artificial intelligence (XAI), identifying post-hoc interpretability as a central enabler of trust, particularly in high-stakes and industrial settings. The value of local explanation methods was further emphasised for their role in promoting transparency and accountability.

Complementary to these perspectives, Ref. [13] benchmark a wide range of explanation methods, providing a quantitative basis for evaluating post-hoc interpretability. In parallel, Ref. [14] highlights that explainability and interpretability are now established as central themes across diverse application domains, reinforcing the relevance of this study’s focus on industrial model analysis.

Initial exploration of dimensionality reduction methods, including Principal Component Analysis (PCA) and Kernel Principal Component Analysis (KPCA), has demonstrated their capacity to detect structural regimes and variance clusters within campaign-unfolded batch data [15]. The present work extends this direction by embedding interpretability metrics within a continuous surrogate surface, thereby enabling systematic investigation of regions characterised by uncertainty, systematic bias, or high model confidence.

Accordingly, a model-agnostic mapping framework is introduced to support the post-hoc analysis of trained machine learning models. Rather than treating interpretability as a collection of isolated local explanations, the proposed approach seeks to identify structured regimes in model behaviour across the input space. To this end, interpretability metrics, such as SHAP values, class probabilities, and residuals, are projected into a low-dimensional latent space using Uniform Manifold Approximation and Projection (UMAP) and subsequently approximated through a continuous surrogate surface. This yields a spatial representation of model behaviour in which regions of confidence, ambiguity, and systematic deviation can be examined in relation to one another.

The resulting decision landscape is therefore not only a visual aid but also a structured representation that supports analysis of model behaviour across the embedding space. In this representation, local patterns may be interpreted as regime-dependent behaviour rather than isolated irregularities, allowing the analyst to distinguish stable regions from transitional or unreliable ones. This perspective supports both exploratory diagnostics and targeted refinement, as selected regions may be traced back to the original input space and examined in relation to process variables and operational history.

This interpretation is supported by recent work on feature-based remaining useful life modelling of electrical resistance heating wires, where post-hoc analysis revealed that predictive behaviour varies systematically across degradation regimes and that uncertainty and error concentrate in transitional phases rather than being uniformly distributed [16]. Importantly, these structures were not captured by aggregate performance metrics, but only became visible through region-wise analysis of model behaviour. Motivated by these findings, the present work treats post-hoc analysis not merely as an explanation, but as a structured approach for identifying regime-dependent reliability in industrial machine learning models.

Accordingly, this study is guided by the following research question: Can embedding interpretability metrics into a continuous low-dimensional representation reveal structured, region-dependent variation in model behaviour that is not observable through aggregate performance metrics or pointwise explanations alone?

To address this question, the proposed framework is evaluated on an industrial case study with the objective of identifying whether stable and transitional regimes in model behaviour can be consistently detected and related to process conditions.

A case study is conducted on Kanthal’s ceramic heating element production data, focusing on the prediction of post-extrusion quality compliance in the final operation. Two patterns are identified: one corresponding to a historical process deviation, and another reflecting a more recent operational fluctuation. Together, these findings illustrate the utility of the framework in bridging data-driven inference with process expertise.

### 1.1. Case Study

This study examines the production of post-extrusion quality compliance at Kanthal’s facility in Hallstahammar, Sweden, where the post-extrusion treatment concludes the sequential manufacture of ceramic electric heating elements used in high-temperature industrial applications.

The process includes 11 distinct operations. Due to confidentiality, the associated features (comprising sensor readings and material properties) have been anonymised and enumerated accordingly, and the operational context has been intentionally generalised to avoid disclosing proprietary details.

**Figure 1:**
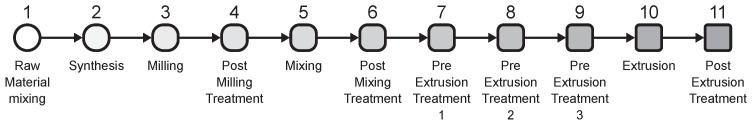
Flowchart visualising the involved operations.

**Figure 2:**
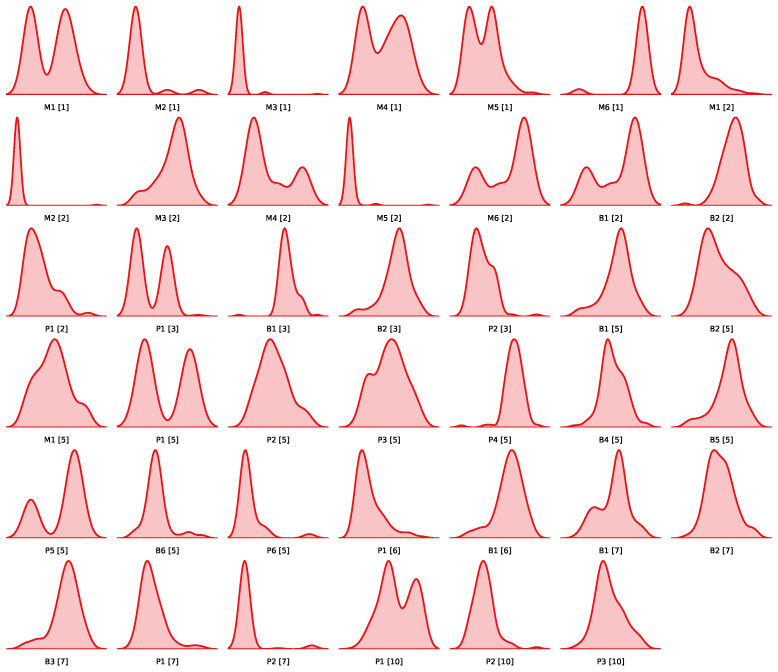
KDE full dataset—displaying all 43 features. M: material properties (e.g., elemental composition); B: batch properties (e.g., weight); P: process properties (e.g., time, temperature).

The data used for modelling are derived from 11 sequential processing steps, beginning with operation 1, raw material mixing, and concluding with operation 11, post-extrusion treatment, and are visualised in Figure 1. Each operation contributes material or sensor-derived information, cumulatively forming a multivariate input space. Figure 2 lists and shows the distribution of each included sensor.

Following operation 1, raw material mixing, the sequence continues with operation 2, synthesis, to initiate precursor reactions, operation 3, milling, for particle refinement, and operation 4, post-milling treatment, as a solvent removal and slurry conditioning stage. Subsequently, operation 5, mixing, ensures thorough homogenisation, while operation 6, post-mixing treatment, performs ceramic body treatment through controlled solidification. This is conditioned through a series of pre-extrusion treatments: operation 7, pre-extrusion treatment 1, operation 8, pre-extrusion treatment 2, and operation 9, pre-extrusion treatment 3. Final shaping occurs during operation 10, extrusion, and the pipeline concludes with operation 11, post-extrusion treatment.

The final step serves as the point at which the process must pass post-extrusion Quality Assurance (QA), forming the basis for the post-extrusion quality compliance used in this study.

A primary machine learning model is trained using upstream input space features to predict post-extrusion quality compliance outcomes. Two prediction formulations are considered.

**Figure 3:**
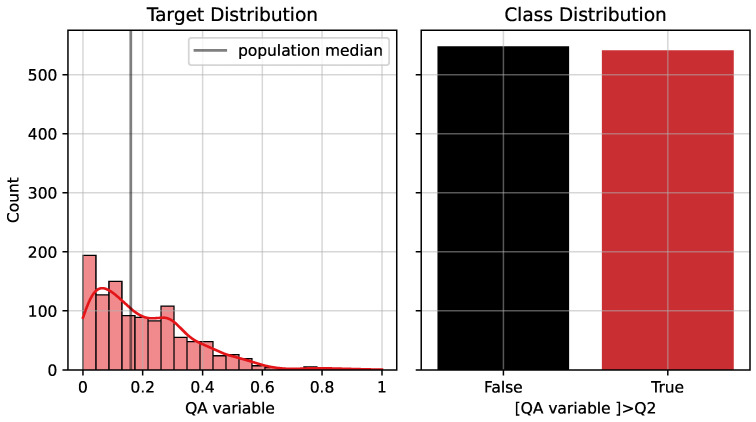
KDE target—displaying post-extrusion quality compliance distribution and class distribution.

In the regression setting, the model estimates the continuous post-extrusion quality compliance (Figure 3, left). The bars show the binned distribution, while the red line represents the KDE of the target distribution. The vertical black line indicates the population median, which is used as the threshold for the classification formulation.

In the classification setting, a binary target is constructed by thresholding the observed QA compliance distribution at its median value (Figure 3, right). Samples above the median are assigned to the positive class, while samples below the median are assigned to the negative class. The black bar represents the number of samples below the threshold, while the red bar represents the number of samples above the threshold. This framing allows both fine-grained prediction and interpretable classification of process outcomes.

Figure 2 and Figure 3 provide visual support for the dataset structure and target distribution, respectively. These figures illustrate the input variable distributions and the framing of the prediction problem across regression and classification settings.

The model is constructed to learn from process conditions observed before the post-extrusion treatment, with the aim of predicting QA compliance outcomes at the final stage. This formulation reflects a realistic industrial scenario in which early detection of non-compliant products is desirable. The selected inputs, extracted before the decision point, ensure that the model respects causal ordering and remains applicable in deployment.

Accordingly, this study investigates whether embedding interpretability metrics into a continuous low-dimensional representation can reveal structured, region-dependent variation in model behaviour that is not captured by aggregate performance metrics.

The contribution of this work lies in the formulation of a model-agnostic post-hoc analysis framework that combines local interpretability metrics, nonlinear embedding, and surrogate-based mapping to support region-wise diagnostics of model behaviour.

Rather than providing pointwise explanations alone, the proposed approach seeks to reveal coherent regions of confidence, ambiguity, and systematic deviation, thereby enabling structured analysis of model generalisation in industrial settings.

While methods such as SHAP and Local Interpretable Model-agnostic Explanations (LIME) provide local explanations of individual predictions, they do not reveal how model behaviour is structured across the input space. Similarly, low-dimensional embeddings enable visual exploration of data structure, but do not directly capture how interpretability metrics vary across regions.

Global interpretability approaches such as permutation feature importance or Partial Dependence Plot (PDP) analysis provide aggregate descriptions of model behaviour, but typically reduce behaviour to feature-level summaries or low-dimensional response curves. While useful for identifying dominant variables or average trends, such methods do not preserve the spatial organisation of model behaviour across the input space. In contrast, the proposed framework constructs a spatially indexed representation of interpretability metrics over the embedding space, enabling analysis of coherent behavioural regions, transitions, and localised regimes within the learned model response.

The proposed approach extends these methods by approximating interpretability metrics as continuous functions over the embedding space. This enables the identification of gradients, transitions, and coherent regions in model behaviour, which are not accessible through pointwise explanations or scatter-based visualisation alone.

The choice of a two-dimensional embedding is not intended to maximise embedding fidelity, but to ensure that the resulting structure remains visually interpretable and directly accessible to domain experts. This facilitates interactive analysis and allows identified regions to be traced back to the original input space, supporting practical interpretation in an industrial context.

The dataset consists of 1082 samples collected over more than a decade of production, where each sample represents a complete production instance. The input space comprises 43 variables derived from material, batch, and process measurements.

The data are partitioned into training and test subsets using a random split ratio of 80/20. All preprocessing steps, including scaling and dimensionality reduction, are fitted exclusively on the training data and subsequently applied to the test set to prevent information leakage.

### 1.2. Background

This section presents key technical foundations underpinning the proposed approach, including model selection, dimensionality reduction, and post-hoc interpretability within industrial contexts.

#### 1.2.1. Support Vector Regression

Support Vector Regression (SVR) is employed as a surrogate model to construct a continuous representation of the selected interpretability metrics over the embedding space. In this role, the objective is not to maximise predictive accuracy, but to approximate the dominant structure of model behaviour in the reduced space.

By learning a mapping from embedding coordinates to the chosen interpretability metric, SVR defines a smooth surface that enables spatial analysis of model confidence, error, or attribution patterns. The *ε*-insensitive loss suppresses minor local fluctuations, while the regularisation parameter *C* controls the trade-off between smoothness and fidelity [17].

This behaviour is desirable in the present context, as the aim is to reveal stable regions, gradients, and transitions in model behaviour rather than to interpolate noise. The resulting surface therefore, acts as a continuous approximation of the interpretability metric, supporting region-wise diagnostics and interpretation.

#### 1.2.2. Uniform Manifold Approximation and Projection

UMAP is used to define a low-dimensional embedding space in which model behaviour can be analysed spatially. The purpose of this transformation is not limited to visualisation, but to construct a latent coordinate system over which interpretability metrics can be organised and approximated.

High-dimensional input data are projected into a two-dimensional space that preserves local neighbourhood structure while retaining aspects of global geometry. This enables the identification of coherent regions and transitional boundaries in model behaviour, which would be difficult to observe directly in the original input space.

A key property of UMAP in this context is its ability to support out-of-sample transformation, allowing unseen observations to be embedded consistently. This ensures that both training and test data can be analysed within the same latent space, supporting assessment of generalisation behaviour.

Within the proposed framework, UMAP therefore serves as the latent coordinate system that underpins region-wise diagnostics and interpretation of model behaviour [18].

#### 1.2.3. Industrial Applications in Post-Hoc Model Evaluation

In dynamic production environments, where auditability and reliability are required, post-hoc model explainability has the potential to enable the industrial deployment of machine learning.

eXplainable Artificial Intelligence (XAI) methods have been adopted in manufacturing contexts to support supervised [19] and unsupervised [20] fault detection. LIME provides local surrogate models to explain individual predictions [11]. SHAP offers feature attributions that are theoretically grounded [21], and which are also consistent, locally accurate, and globally aggregatable [12].

Model-specific implementations such as Tree SHAP have been developed to reduce computational overhead. Complementary tools, including PDP and Individual Conditional Expectation (ICE) curves, are commonly employed to support feature-level interpretability.

Recent reviews have emphasised critical XAI desiderata in industrial contexts, including fidelity [10], stability [22], and computational efficiency [23]. Other studies have highlighted the need to contextualise explainability within concerns of robustness, causality, and data integrity, particularly in time-sensitive and high-reliability domains such as predictive maintenance and IIoT systems [24].

While post-hoc feature attributions offer localised insights, they are typically limited to pointwise interpretations. To reveal higher-order structure in model behaviour and data topology, recent work in smart manufacturing has explored topology-based representations of process variation [25].

Inspired by this direction, the present work leverages UMAP-based embeddings to project local interpretability metrics into a global low-dimensional space. This enables region-wise diagnostics and supports model-level generalisation assessment.

In contrast to maintenance-oriented explainability pipelines, the present work combines SHAP-based local attributions with UMAP-derived global embeddings to visualise and assess generalisation performance. This enables targeted diagnostics not only of feature contributions but also of broader model behaviour across the embedding space.

Recent applications confirm that UMAP projections can amplify early-stage deviations in high-dimensional time series data, improving sensitivity to fault transitions before deterioration becomes evident in conventional indicators [26]. Such diagnostics align with the dual notion of trust described by Ribeiro et al. [27], in which explaining individual predictions supports confidence in specific outputs, while analysis of representative samples builds trust in model behaviour overall.

While methods such as SHAP and LIME offer valuable insights into model behaviour, their limitations must be acknowledged. These include low fidelity, instability, and restricted applicability for demonstrating legal or regulatory fairness, particularly in high-stakes environments [28].

## 2. Methodology

**Figure 4:**
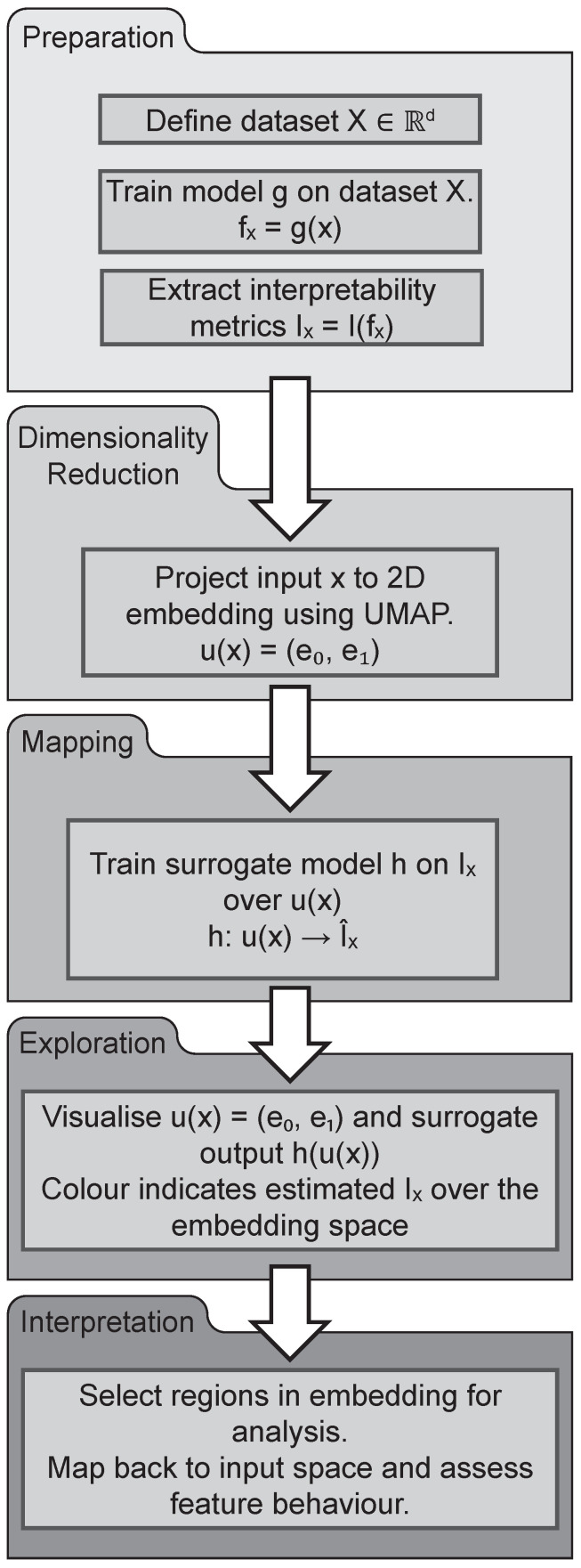
Schematic of the post-hoc model analysis framework, organised into five phases: (i) Preparation; (ii) Dimensionality Reduction; (iii) Mapping; (iv) Exploration; and (v) Interpretation.

The proposed approach is organised into five conceptual phases, as illustrated in Figure 4. The aim is to construct a structured decision landscape over a low-dimensional embedding space, enabling post-hoc analysis of generalisation behaviour and uncertainty.

This rationale resonates with recent advances emphasising not only local post-hoc explanations but also the stability and actionability of explanatory procedures [29]. Our framework extends these ideas by integrating interpretability metrics into embedding-informed surrogate surfaces, thereby enabling region-wise diagnostics and selective modelling in evolving industrial contexts.

The proposed framework operates on interpretability metrics, which act as observable proxies for model behaviour at the level of individual predictions. These metrics do not describe the input data directly, but instead characterise how the trained model responds to it. Depending on the modelling objective, different interpretability metrics capture complementary aspects of behaviour. Class probabilities reflect model confidence, residuals capture prediction error and bias, and SHAP values describe the contribution of input features to individual predictions. Despite their differences, all such metrics can be interpreted as scalar fields defined over the input space.

While interpretability metrics such as SHAP values or residuals provide local, pointwise descriptions of model behaviour, their direct visualisation in the embedding space remains discrete and often difficult to interpret in terms of coherent structure.

To address this, a surrogate SVR model is used to construct a continuous approximation of these metrics over the embedding space. This enables the analysis to move beyond individual observations toward the identification of gradients, transitions, and spatially coherent regions in model behaviour.

In contrast to direct scatter-based visualisations, the resulting surface supports interpretation of model behaviour as a structured field, where regions of confidence, ambiguity, and systematic deviation can be examined in relation to one another. This provides a basis for region-wise diagnostics that are not readily accessible from pointwise explanations alone.

Preparation

The process begins with a dataset $X\in R^{d}$ defined in the original input space. A primary estimator $g:R^{d}\to R$ is trained on *X*, producing predictions $f_{x}=g\left(x\right)$. From these outputs, a set of interpretability metrics is extracted, denoted $I_{x}=I\left(f_{x}\right)$. These may include class probabilities, residuals, or SHAP values, depending on the task and model architecture.

Dimensionality Reduction

To enable spatial organisation and visualisation, each input sample is embedded into a two-dimensional embedding space using UMAP. This transformation is written as $u\left(x\right)=\left(e_{0},e_{1}\right)$, where $u:R^{d}\to R^{2}$ preserves both local and global structure.

Mapping

A surrogate SVR model $h:R^{2}\to R$ is trained to regress selected interpretability metrics onto the UMAP coordinates. The resulting continuous surface h(u(x)) defines the decision landscape, revealing spatial patterns in model confidence or misclassification.

Exploration

The decision landscape is visualised, and features such as gradients, clusters, and decision boundary analysis are identified. Regions of interest are determined based on a combination of metric-based criteria and spatial structure in the embedding space. In practice, this involves applying thresholds on the selected interpretability metrics (e.g., class probability or residual magnitude) to isolate subsets of interest, followed by analysis of their distribution in the embedding space.

Regions are operationally defined as subsets of samples that simultaneously satisfy (i) a specified threshold on the interpretability metric and (ii) localisation within a bounded area of the embedding space. This ensures that extracted regions reflect both similar model behaviour and proximity in the learned representation.

Visual inspection may be used to guide the initial identification of candidate regions; however, the final definition is based on explicit filtering criteria in both metric and embedding coordinates. This ensures that region extraction is reproducible and not solely dependent on subjective interpretation.

Interpretation

Selected regions are mapped back to the input space for further interpretability metric analysis. Feature distributions are examined and interpreted in collaboration with domain experts. The insights derived from this process may guide selective modelling, model refinement or operational change.

In this way, interpretability is treated not only as a diagnostic tool but also as a form of safety, consistent with perspectives that frame explanation as a safeguard for reliable and accountable data mining [14].

### 2.1. Pipeline Configuration

The configuration of the decision landscape pipeline combines domain-informed design choices with flexible, robust tools for model training and hyperparameter search. All estimators and sampling procedures are implemented using the scikit-learn library [30], which provides a widely used and well-tested framework for machine learning in Python. Notable exceptions include the use of UMAP [18] for nonlinear dimensionality reduction and SHAP values [12] for local feature attributions, both of which integrate into the pipeline while extending its capabilities beyond core scikit-learn functionality. The following paragraphs describe the configuration parameters for interpretability metrics, the definition of parameter ranges and sampling distributions, and the search strategy used to ensure representative coverage of model behaviours.

Interpretability Metrics

The target surface is formed using one or more interpretability metrics. These may include class confidence, directionally signed residuals, absolute residual magnitudes, or local feature attributions such as SHAP values.

Parameter Ranges and Sampling

To accommodate varying levels of complexity within the decision landscape, a range of hyperparameter values is sampled. These are denoted using U to represent uniform distributions. U[a, b] denotes a continuous uniform distribution on the interval [a, b]. U{a, a+1, …, b} denotes a discrete uniform distribution over integers. The domain of each parameter is inferred from context within the pipeline configuration. This sampling procedure enables broad exploration of the latent structure in the embedded model behaviour.

Random Grid Search for Hyperparameter Tuning

Random grid search is employed as a pragmatic strategy for hyperparameter exploration within the defined ranges. Unlike exhaustive grid search, which evaluates all possible parameter combinations, random grid search samples a fixed number of configurations at random. This enables broader coverage of the parameter space with reduced computational cost. The approach is particularly effective when only a subset of hyperparameters significantly influences model performance. It mitigates the curse of dimensionality inherent in high-dimensional grids [31]. By combining domain-informed parameter ranges with stochastic sampling, the method balances thoroughness and efficiency, facilitating the identification of robust configurations that generalise well across the industrial dataset.

Interpretation of the Information Landscape

The information landscape visualises the distribution of the mapped output variable within a reduced two-dimensional space. The horizontal and vertical axes correspond to the reduced dimensions, denoted $e_{0}$ and $e_{1}$, respectively. These coordinates are obtained through dimensionality reduction and preserve relevant variance from the original high-dimensional input space.

Each point in the landscape represents an individual observation. Two distinct marker symbols are used to distinguish the data partitions: upward-pointing triangles denote samples from the training dataset, while downward-pointing triangles denote samples from the testing dataset. This distinction enables an assessment of the mapping’s generalisation performance by comparing how mapped values behave for both seen and unseen data.

The colour of each marker encodes the true value of the mapped output variable for that observation. This colour mapping facilitates visual comparison between the local values in the embedding space and the underlying reference data. The colour scale is indicated by an accompanying colourbar, which defines the value range across the entire landscape. Additionally, solid contour lines are superimposed onto the landscape to indicate the distribution of mapped values. Each contour line represents a decile (10th quantile) of the mapped output. These isolines highlight regions with similar mapping outputs and make local gradients and transition zones within the surface easier to interpret.

**Figure 5:**
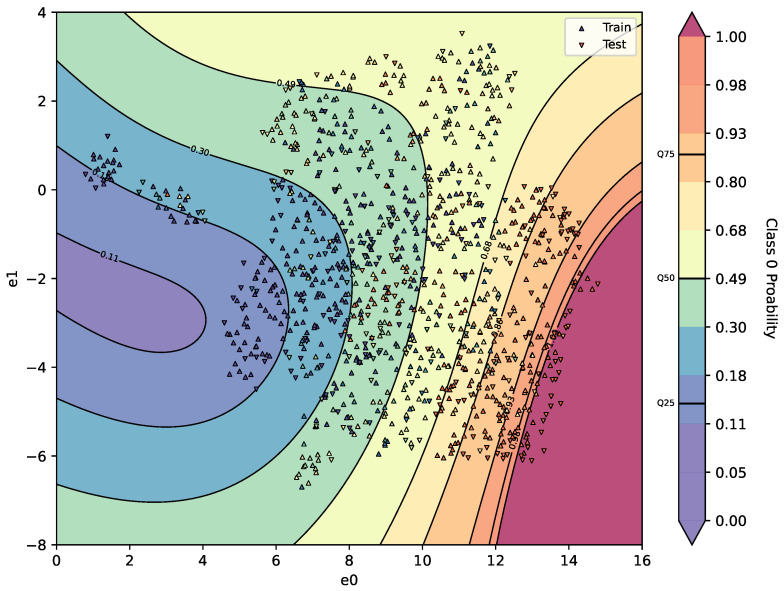
Pattern 1—UMAP-informed surrogate response surface—Class 1 Probability. The marker and surface color shows the value of the target interpretability metric and the colorbar indicates the value range across the entire landscape. The contour lines represent deciles of the mapped output.

The interpretation described here applies directly to the information landscape visualisations presented in Figure 5.

Robustness and Stability

The training and parameter setup described above ensures that each analysed configuration is reproducible and that observed structures are not the result of test-set tuning or manual adjustment. However, reproducibility of a single configuration should be distinguished from robustness across configurations.

In the present framework, robustness refers to the consistency of identified regions under variation of embedding and surrogate parameters, such as UMAP neighbourhood size, minimum distance, distance metric, and SVR regularisation. The present study primarily establishes a controlled and reproducible analysis procedure, while systematic quantification of structural stability across the full parameter space is left for future work.

Accordingly, the identified regions are interpreted as reproducible structures under the specified configuration, rather than as formally invariant partitions of the input space.

Exploratory variation of embedding and surrogate parameters indicated that stable regions such as pattern 1 persist across configurations, whereas transitional regions such as pattern 2 exhibit higher sensitivity, consistent with their interpretation as less well-defined regimes.

### 2.2. Training and Parameter Setup

The training procedure is designed to ensure reproducibility, separation of concerns, and to avoid information leakage between modelling stages. The framework is structured as a sequence of independent steps, where each transformation is fitted exclusively to training data and subsequently applied to unseen data.

All stochastic components of the pipeline, including data splitting, hyperparameter sampling, and embedding initialisation, are controlled through fixed random seeds to ensure reproducibility. All model selection and parameter tuning are conducted exclusively on the training data, and the held-out test set is not used at any stage of model fitting or configuration. The primary estimator is trained using cross-validated random search over predefined hyperparameter ranges, following standard practice in supervised machine learning. The resulting model is evaluated on the test set without further adjustment, and no parameters in the embedding or surrogate mapping stages are tuned based on test performance or visual inspection of the resulting structures. This separation ensures that observed patterns arise from the learned model behaviour rather than from iterative adjustment of the analysis pipeline.

Following model training, interpretability metrics are computed for each observation. For regression tasks, the interpretability metric is defined as the signed residual $I_{x}=f_{x}-y$, where $f_{x}$ is the model prediction and *y* the true target value. For classification tasks, the interpretability metric is defined as the predicted probability of the true class $I_{x}=p_{y}$. These metrics serve as observable proxies for model behaviour and form the basis for subsequent analysis.

Dimensionality reduction is performed using UMAP. The embedding is fitted exclusively on the training data, thereby defining a latent coordinate system u(x). Test samples are subsequently projected into this space using the learned transformation, ensuring that no information from the test set influences the embedding.

A surrogate SVR model is then trained to approximate the selected interpretability metric as a function of the embedding coordinates. The surrogate is fitted using only the training data, mapping $u\left(x\right)\mapsto I_{x}$, and is subsequently evaluated on both training and test samples to assess generalisation of the mapping.

Hyperparameters for both UMAP and SVR are selected using random search within predefined ranges. These ranges are specified a priori and are chosen to span both low- and high-complexity configurations. Parameter configurations are sampled rather than manually adjusted. Optimisation is performed with respect to reconstruction of the interpretability metric, rather than visual separability or the appearance of specific structures in the embedding.

Under this setup, observed structures in the decision landscape arise from the interaction between the data, the trained model, and the predefined parameter ranges, rather than from manual tuning or post-hoc adjustment.

This design ensures that the resulting analysis reflects consistent and repeatable behaviour of the modelling pipeline, providing a principled basis for interpreting region-wise variation in model performance. While the exact geometric representation may vary under different parameter samples, the analysis focuses on structural regions that are consistent across configurations rather than on pointwise alignment.

## 3. Results & Discussion

This section presents the results of applying the proposed post-hoc analysis framework to the industrial case study. The objective is not only to identify distinct regions in the decision landscape, but to assess whether model behaviour is structured into regimes with differing reliability, uncertainty, and interpretability.

Rather than treating extracted patterns as isolated anomalies, they are interpreted as manifestations of underlying generalisation structures. That is, the extracted patterns are interpreted as structured variations in model behaviour, rather than as definitive or exhaustive partitions of the input space. In this view, model behaviour is not assumed to be uniform across the input space, but instead varies systematically, with certain regions exhibiting stable predictions while others concentrate ambiguity or deviation.

The analysis therefore, focuses on where the model behaves consistently, where ambiguity concentrates, and how these regions relate to process conditions and temporal evolution.

### 3.1. Pattern 1—Major Trend

Pattern 1 is identified using class probabilities from the primary classifier as the target interpretability metric, combined with a simple SVR-based model-agnostic mapping. The resulting decision landscape is shown in Figure 5, with parameters detailed in Table 1. The reported configuration represents a high-performing reproducible sample obtained through stochastic parameter search within the predefined ranges described in Section 2.2, rather than manual tuning for visual separability.

In the plot, the horizontal and vertical axes correspond to the UMAP embedding dimensions $e_{0}$ and $e_{1}$, respectively. Samples are shown as markers: upward triangles represent training data, and downward triangles denote test samples. The background shading visualises the predicted class probability surface h(u(x)) derived from the SVR.

The extracted region forms a coherent high-confidence regime in the embedding space, suggesting consistent model behaviour within this subset. This suggests that the model appears to have learned a stable and well-represented portion of the process space, rather than merely identifying a cluster of similar samples.

In this region, predictions are both confident and accurate, reflecting a strong alignment between the learned mapping and the underlying data structure. As such, pattern 1 is interpreted as a stable regime of model behaviour, in which generalisation performance is locally reliable.

The colour intensity reflects the model’s classification confidence. Red indicates a strong likelihood of being classified as compliant (i.e., passing post-extrusion quality compliance). Blue indicates a strong likelihood of being classified as non-compliant. Yellow denotes model uncertainty near the decision boundary analysis. A high-probability region emerges in the lower-right quadrant of the embedding. This area is dominated by samples predicted as non-compliant with high certainty. To investigate further, a threshold is applied to extract these samples based on their coordinates and predicted probability. This subset is labelled as P1; the remainder is denoted Rest.

**Figure 6:**
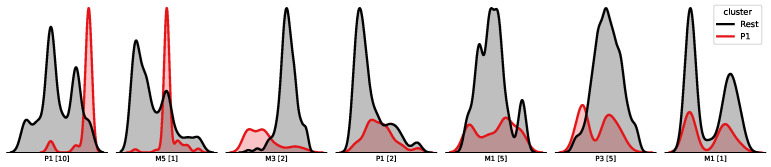
Pattern 1—KDE.

To interpret the extracted region, the P1 and Rest subsets are visualised in Figure 6 across top-ranked features based on shapley feature importance. Three distinct filtering conditions emerge: Feature P1[10] identifies samples exhibiting a peak value in a specific process variable during the penultimate operation (visible as a concentration on the right edge of the distribution). Feature M3[1] captures samples with a mid-range material property measured during the first operation (a clear congregation of samples appears in the centre of the distribution). Feature M3[2] includes only samples where a particular property from the second operation does not exceed a defined lower-bound threshold (the distribution is clearly separated from its counterpart around the centre).

To assess the classifier’s generalisation performance across distinct subregions of the data landscape, accuracy was evaluated separately for the P1 region and the remaining population, as shown in Table 2. The baseline model achieves 90% accuracy across the entire population. When scored only on the P1 region, the classifier achieves 97% accuracy on 17% of the population, outperforming the baseline for that subset. The remaining samples yield an accuracy of 78%. These results provide quantitative support for the observed regional variation in model behaviour.

These differences provide quantitative support for the observed regional variation in model behaviour.

**Figure 7:**
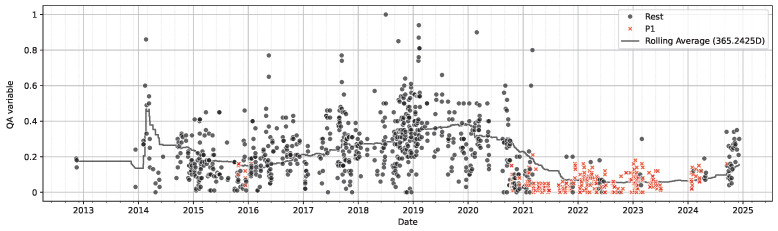
Pattern 1—Target over time.

To validate the operational significance of pattern 1, extracted samples were plotted over time (Figure 7). Discussion with domain experts revealed that this segment corresponds to a known process shift related to improvement actions initiated around early 2021. Notably, this behaviour does not persist consistently throughout 2021, indicating a shift and motivating the investigation of inconsistencies.

### 3.2. Pattern 2—Recent Fluctuation

**Figure 8:**
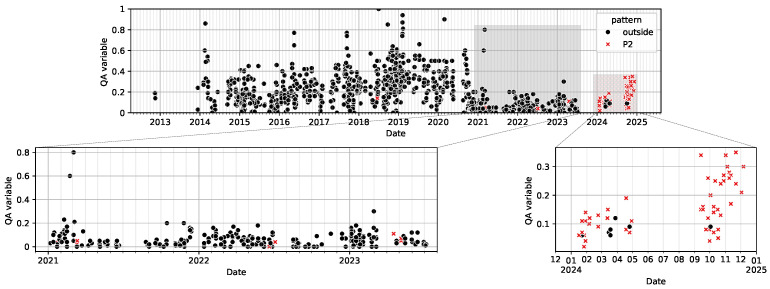
Prediction target plotted over time with rolling yearly average, the samples covered by Pattern 2 are highlighted. Top: Entire span. Bottom-left: Zoom into 2021–2025. Bottom-right: Zoom into 2024-06–2025.

Pattern 2 is motivated by an observed increase in the post-extrusion quality compliance after mid-2024 (Figure 8). Unlike pattern 1, this region cannot be isolated using the original low-complexity configuration, as post-2024 samples embed diffusely into the slope of the existing decision landscape. That is, the model fails to separate these samples both locally and globally.

**Figure 9:**
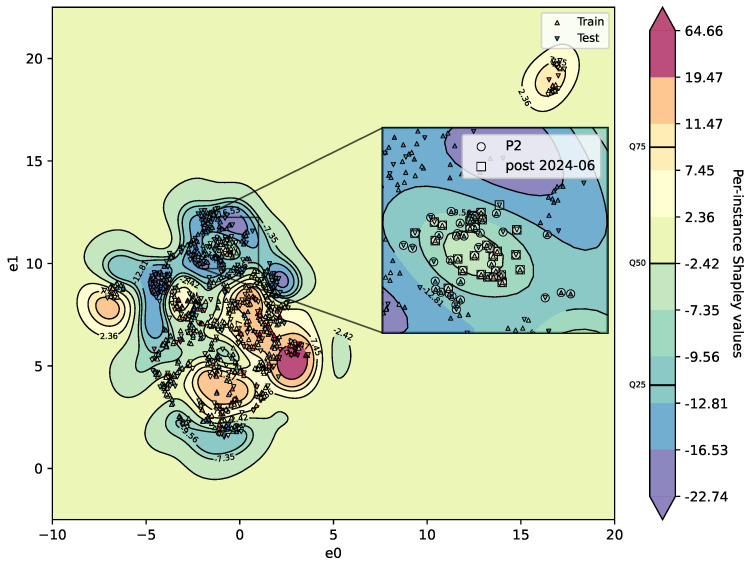
Pattern 2—UMAP-informed surrogate response surface. Circle markers indicate samples identified as P2, while square markers represent samples belonging to the post-2024-06 region. The marker and surface color shows the value of the target interpretability metric and the colorbar indicates the value range across the entire landscape. The contour lines represent deciles of the mapped output.

To isolate pattern 2, the model-agnostic mapping is reconfigured with higher complexity and uses per-instance SHAP values as the target interpretability metric. The parameter range and resulting hyperparameters, along with the decision surface, are shown in Table 3 and Figure 9, respectively. An “island” emerges in the embedding space that includes the post-2024 fluctuation samples as well as a limited number of earlier samples.

The reported configuration represents a high-performing reproducible sample obtained through stochastic parameter search within predefined parameter ranges, rather than manual tuning to isolate a desired structure or maximise visual separability.

This region corresponds to samples increasingly likely to be classified as non-compliant. While the island is well separated locally, a small number of earlier samples are embedded within it, indicating that the region is coherent but not fully isolated in the global structure.

In contrast to pattern 1, the identified region represents a less separable and more ambiguous portion of the decision landscape. The inability to isolate this region under a lower-complexity configuration indicates that the observed structure in model behaviour is not readily captured by simpler representations.

Following reconfiguration, the emergence of a distinct region suggests the presence of a transitional regime, characterised by increased sensitivity to feature interactions and reduced model stability. In this region, model behaviour is less consistent, indicating a mismatch between the learned representation and the underlying process variation.

The contrast between pattern 1 and pattern 2 indicates that model performance is not uniform across the input space, but depends on the local structure of the data. While pattern 1 represents a stable regime in which the model generalises reliably, pattern 2 reflects a transitional regime requiring higher representational complexity.

This suggests that a single global model may be insufficient to capture all observed behaviours, and that region-specific strategies, such as selective modelling or targeted refinement, may provide a more effective approach. In this context, the decision landscape serves as a diagnostic tool for identifying regions where such interventions may be required.

**Figure 10:**
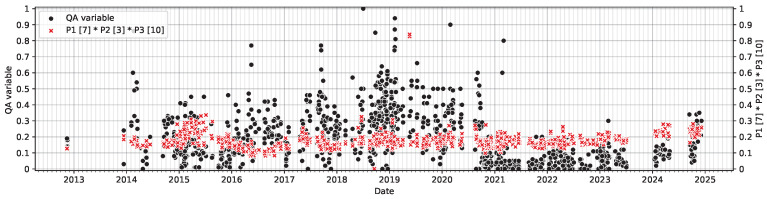
Prediction target plotted over time with rolling yearly average as well as composite feature.

To further characterise this regime, a regression is performed within pattern 2 using third-order interaction terms. The strongest composite feature involves variables from operation 7, pre-extrusion treatment 1, 3, milling, and operation 10, extrusion. Its time series is shown in Figure 10, showing alignment with the observed fluctuation. This composite interaction is consistent with domain expert interpretation and supports ongoing process investigation.

To assess how well the identified region captures the observed temporal fluctuation, the overlap between the pattern 2 region and the post-2024 period was evaluated, as summarised in Table 4.

The identified pattern 2 region captures nearly all samples belonging to the post-2024 regime, while still containing a smaller subset of earlier observations. This indicates that the region aligns strongly with the observed temporal fluctuation, although the underlying structure is not completely separable from historical process behaviour. The results therefore, support the interpretation of pattern 2 as a transitional regime associated with a recent process shift, rather than a random variation in the embedding.

A brief review of the samples captured within the focus region further reveals strong separation in parameters B2[5] and M3[2], indicating that the identified regime is associated with distinct process- and material-related characteristics.

### 3.3. Interpretation of the Framework and Implications

The results indicate that model behaviour is structured across the embedded representation rather than uniformly distributed. In particular, Pattern 1 corresponds to a stable regime in which the model generalises reliably, while Pattern 2 reflects a transitional regime characterised by increased ambiguity and sensitivity to feature interactions.

Importantly, these structures are not directly observable through aggregate performance metrics or standard pointwise explanations alone. While SHAP values and residuals describe behaviour at the level of individual observations, they do not reveal how this behaviour is organised across the input space. By embedding and approximating these metrics as a continuous decision landscape, the proposed framework enables the identification of coherent regions that can be related to process conditions and temporal shifts, as demonstrated in the case study. This provides a structured basis for interpreting model behaviour beyond isolated explanations.

From an operational perspective, the identified regions provide a basis for targeted intervention. Stable regions, such as pattern 1, may be prioritised for deployment or trusted decision support, as model behaviour is consistent and reliable. In contrast, transitional regions, such as pattern 2, highlight areas where model predictions are less stable and warrant further investigation.

In practice, such regions may motivate actions, including additional data collection, refinement of feature representations, or the introduction of region-specific decision rules. Transitional regions exhibiting unstable or ambiguous behaviour may additionally be integrated into industrial monitoring workflows as triggers for expert review, targeted retraining, or process investigation. Conversely, stable regions may support higher-confidence deployment and automated decision support under monitored operating conditions. In this way, the framework supports not only post-hoc interpretation, but also operational decision-making regarding model usage, monitoring, and refinement.

## 4. Conclusions

This work introduced a post-hoc analysis framework for analysing trained machine learning models through embedding-informed surrogate representations of interpretability metrics. The objective was to assess whether model behaviour can be analysed as structured variation across the input space, rather than through aggregate performance metrics alone.

The results show that model behaviour can be organised into distinct regions characterised by differences in predictive reliability and separability. In the presented case, one region corresponds to stable and well-represented process conditions, while another reflects a transitional regime associated with increased ambiguity and sensitivity to feature interactions. These findings indicate that model performance is not homogeneous, but varies systematically across the data.

The proposed framework enables this structure to be observed by combining local interpretability metrics with low-dimensional embedding and continuous surrogate approximation. This allows model behaviour to be analysed in relation to process conditions and temporal variation, providing a structured basis for post-hoc interpretation beyond pointwise explanations.

At the same time, the framework does not provide formal guarantees of uniqueness or stability of the identified regions, and the results depend on the chosen embedding and surrogate configuration. The analysis should therefore be understood as a reproducible and structured diagnostic approach, rather than a definitive partitioning of the input space.

Future work will focus on validating the framework across additional industrial datasets and process domains to assess its generality. In addition, systematic sensitivity analysis of embedding and surrogate parameters will be conducted to quantify the stability of identified regions. This includes evaluating whether structurally similar behavioural regions emerge consistently across multiple datasets and industrial contexts, thereby enabling assessment of robustness beyond a single process configuration. Finally, methods for automated region extraction and quantitative validation will be explored to reduce reliance on visual interpretation and improve reproducibility. 

## Figures and Tables

**Table 1:** Pattern 1 Parameter Values.

Step	Parameter	Range	Value
svr	C	U[0.1, 2.1]	1.75
svr	epsilon	U[0.1, 0.3]	0.202
svr	gamma	scale	scale
umap	metric	euclidean	euclidean
umap	min_dist	U[0.5, 0.99]	0.542
umap	n_neighbors	U{30, …, 99}	30

**Table 2:** Pattern 1 selective modelling results.

Group	Accuracy	Coverage
Filter-selected samples	97%	17%
Original (all samples)	90%	100%
Remaining samples	78%	83%

**Table 3:** Pattern 2 Parameter Values.

Step	Parameter	Range	Value
svr	C	U[1, 21]	20.6
svr	epsilon	U[0.05, 0.2R]	0.0673
svr	gamma	[‘*scale*’, ‘*auto*’]	auto
umap	metric	[‘*euclidean*’	cosine
		,‘*cosine*’]	
umap	min_dist	U[0.2, 0.8]	0.206
umap	n_neighbors	U{15, …, 49}	16

**Table 4:** Overlap between Pattern 2 region and post-2024 temporal regime.

Group	Post-2024	Pre-2024
All samples	32	1029
pattern 2 region	31	30

## Data Availability

The data presented in this study are not publicly available due to confidentiality constraints imposed by the industrial partner.
